# Clustering of Risk Factors With Smoking Habits Among Adults, Sousse, Tunisia

**DOI:** 10.5888/pcd10.130075

**Published:** 2013-12-19

**Authors:** Jihene Maatoug, Imed Harrabi, Sonia Hmad, Mylene Belkacem, Mustafa al’Absi, Harry Lando, Hassen Ghannem

**Affiliations:** Author Affiliations: Imed Harrabi, Sonia Hmad, Mylene Belkacem, Hassen Ghannem, Department of Epidemiology, University Hospital Farhat Hached, Sousse, Tunisia; Mustafa al’Absi, Duluth Medical Research Institute, University of Minnesota, USA; Harry Lando, Department of Epidemiology and Community Health, University of Minnesota, USA.

## Abstract

**Introduction:**

In Tunisia, few studies have assessed the association between tobacco use and other lifestyle risk factors for chronic disease (eg, unhealthy diet, physical inactivity). We studied 1,880 adults to determine the association between tobacco use and other lifestyle risk factors in Tunisia.

**Methods:**

This study was part of an assessment of the prevalence of chronic disease risk factors in a community-based trial conducted in 2009 to implement a chronic disease prevention program. The study population was randomly selected from 3 districts of the region of Sousse. The questionnaires were administered by personal interview and included the assessment of tobacco use and other chronic disease risk factors such as unhealthful diet habits and physical inactivity.

**Results:**

Of the 1,880 study participants, 64% were women. The mean age of the participants was 37.9 (standard deviation, 13.5 y). The prevalence of tobacco use in our population was 50.4% for men and 3.1% for women. Among men, the proportion of alcohol consumption was significantly higher among smokers (25.3% vs 5.7% [*P *<.001]). Smokers consumed fewer fruits and vegetables and more high-fat, high-salt, and high-sugar foods than did nonsmokers. There was no significant difference between male smokers and nonsmokers regarding physical activity (*P* = .36).

**Conclusion:**

Physical activity and dietary characteristics may be important areas for physicians to assess during smoking-cessation interventions.

## Introduction

Approximately 1.3 billion people smoke worldwide and, consequently, 5.4 million people die of diseases caused by tobacco use each year (1). Tobacco use is now ranked as the world’s leading killer because it is a risk factor for 6 of the 8 leading causes of death worldwide (1). Although tobacco use is steadily declining in several developed countries, smoking prevalence and cigarette consumption are increasing in developing countries (2). It is projected that up to 1 billion people will die because of tobacco use during this century, 80% of whom will be in developing countries (1). Tunisia has a high prevalence of tobacco use, but data on the association between tobacco use and other lifestyle risk factors are lacking for the Tunisian population (3).

Studies show that nonsmokers have a healthier lifestyle than do smokers as measured by dietary habits, physical activity, and alcohol consumption (4–7). Concerning dietary habits, smokers tend to drink more coffee and alcohol (5,6) and consume fruits, vegetables (7), brown bread, and skimmed milk less often than do nonsmokers (6). Smoking is correlated with consuming a higher percentage of energy from fat (8) and lower intakes of selected antioxidants than is nonsmoking (9). The prevalence of hypertension (5), elevated cholesterol (5), and elevated triglycerides (8,9) is higher for smokers than for nonsmokers and so is the prevalence of inactivity (10). Smoking cigarettes is associated with a change in dietary habits that may contribute to higher risk of coronary vascular disease and other disorders for smokers than for nonsmokers (4). Physical activity is another concern; current smokers are significantly more likely than those who never smoked to be physically inactive (5,10). The prevalence of performing strenuous physical activity at least 1 hour a week is lower among current smokers than among both male and female nonsmokers and former smokers (4). Several studies show that smokers have a lower body mass index (BMI) than do nonsmokers and that the prevalence of obesity is higher among nonsmokers (11,12). Despite their lower BMI, smokers have higher rates of illness and death than nonsmokers have (13). On the other hand, there is a strong association between smoking and mental disorders (14). Current tobacco use is associated with anxiety and affective disorders (15).

Several studies of the lifestyle habits and mental health of smokers worldwide (6,7,14,16) show that smokers consume more fats and alcohol, less fruit, vegetables, and fiber, and are more anxious than nonsmokers (15,16). In Tunisia, little is known about the association between tobacco use and other chronic disease risk factors. Knowing the characteristics of smokers will allow public health experts to adapt interventions for tobacco prevention and lifestyle modification. We conducted this study to determine the association between tobacco use and other lifestyle factors among adults in Tunisia.

## Methods

The region of Sousse is composed of 15 delegations with 544,413 inhabitants and 124,519 households. We selected 3 delegations to participate to our study on the basis of feasibility and sociodemographic characteristics: Sousse Jawhara, Sousse Riadh, and Msaken. These delegations comprised 213,376 inhabitants and 50,058 households. We used cluster sampling to select 1,000 households from 16 randomly selected districts. Questionnaires were administered only to adults aged 18 to 65 years. Data collectors contacted each selected household at their home to ask them to participate in the study. If they found nobody at home at the first contact, they returned to the same home on the weekend. Of the 2,565 adults selected, 73.3% participated in data collection. 

The questionnaire used in this study was prepared by the Oxford Health Alliance for the Community Intervention for Health Project to evaluate smoking, diet, and physical activity habits among adults. We translated this questionnaire to Arabic and pretested it before use.

Trained investigators administered the questionnaires by personal interview. Height and waist circumference were measured to the nearest centimeter and weight to the nearest half-kilogram. BMI was calculated as body weight in kilograms divided by the square of height in meters. Obese people were those who had a BMI of 30 or more (17). Abdominal obesity was defined as waist circumference higher than 102 cm among men and higher than 88 cm among women (18). Blood pressure was measured by using an automated sphygmomanometer with subjects in a seated position, and the mean of 2 repeated measurements was recorded.

We asked participants, “Do you currently smoke any tobacco products, such as cigarettes, cigars, or pipes?” *Smokers* were the participants who responded yes to this question. *Ex-smokers* were people who responded no to this question and yes to the question “If you are not a current smoker, have you ever smoked daily (almost every day for at least 1 year)?” *Never smokers* were people who responded no to both questions.

Health status was coded as good if participants responded *good, very good*, or *excellent* to the question “How would you assess your present state of health?” and coded as not good if they responded *very poor, poor, *or* average*. Two questions assessed emotional status: “During the last month, have you often been bothered by feeling down, depressed, or hopeless?” and “During the last month, have you often been bothered by little interest or pleasure in doing things?” The participants answered yes or no. The question “Have you been feeling tense, stressed or under a lot of pressure during the past month (30 days)?” was coded no if the participant answered “not at all” and coded yes if the participant answered “somewhat but not more than is usual for people in general.” The answer was coded yes if the participant responded “yes, more than is usual for people in general” or “yes, my life is almost unbearable.”

Physical activity was evaluated by asking these 4 questions: 1) “During the past 7 days, on how many days did you do vigorous physical activities?”; 2) “How much time did you usually spend doing vigorous physical activities on 1 of those days?”; 3) “During the past 7 days, on how many days did you do moderate physical activity?”; and 4) “How much time did you usually spend doing moderate physical activities on 1 of those days?”

Statistical analysis was performed using SPSS version 10.0 (SPSS Inc, Chicago, Illinois). Data are presented as frequencies, means, and standard deviations. The χ^2^ test was used to compare percentages between smokers and nonsmokers. Significance was set at .05.

## Results

Our study population comprised 1,880 adults (64% women). The mean age of the participants was 37.9 (standard deviation [SD], 13.5) years with a significant difference between men and women (37 [SD, 14.1] years for men and 38.4 [SD, 13.1] for women, *P* = .03).

The prevalence of tobacco use in our population was 50.4% among men and 3.1% among women. Most smokers were daily smokers: 93.8% of men and 94.4% of women smokers. The proportion of men who had never been smokers was 36.3% and of women, 96.1% (*P *<.001). The proportion of men who were former smokers was 13.3% and of women, 0.8% (*P *<.001).

The mean age of daily smoking initiation was 18.53 (SD, 5.47) years for men and 18.29 (SD, 5.61) for women smokers. The mean number of cigarettes smoked per day was 21.74 (SD, 11.45) cigarettes among men and 18.09 (SD, 11.67) among women smokers. There was no significant difference by sex between the mean age of smokers and nonsmokers (Table 1).

**Table 1:** Sociodemographic Characteristics of 1,880 Adult Smokers and Nonsmokers by Sex in Sousse, Tunisia, 2009

Characteristic	Men (n = 677)		*P* Value	Women (n = 1,203)		*P* Value
**Smoking status, n (%)^a^**						
Smoker	341 (50.4)		NC	37 (3.1)		NC
Nonsmoker	336 (49.6)	NC		1,165 (96.9)	NC	
**Mean age, y (SD)**						
Smoker	37.15 (13.6)		.74	38.86 (11.74)		.82
Nonsmoker	36.79 (14.6)			38.38 (13.17)		
**Have an occupation, n (%)**						
Smoker	311 (91.7)		.95	24 (64.9)		<.001
Nonsmoker	305 (91.9)			406 (35.2)		
**Not married, n (%)**						
Smoker	148 (43.5)		.69	10 (27.0)		.81
Nonsmoker	149 (45.0)			292 (25.3)		
**Primary school education or less, n (%)**						
Smoker	81 (23.9)		.01	10 (27.0)		.06
Nonsmoker	53 (15.9)			492 (42.7)		

Abbreviations: NC, not calculated; SD, standard deviation.

a Comparing male and female smokers,* P* <.001.

Among women, education level did not differ by smoking status (27% of smokers had only a primary education; 42.7% of nonsmokers had only a primary education) (Table 1). Among men, no significant difference was found according to employment or marital status. Male smokers had a lower level of education than did nonsmokers (*P* = .01) (Table 1). The proportion of women who have an occupation was significantly higher for smokers (64.9% vs 35.2%, *P *<.001) (Table 1).

Among men, the proportion of smokers who assessed their health status as good was significantly higher than the proportion of nonsmokers (74.5% vs 66.8%, *P* = .04). Yet both men and women smokers were more likely than nonsmokers to indicate feeling depressed and having little pleasure in doing things during the previous month. However, there was no significant difference between smokers and nonsmokers concerning feeling under a lot of pressure for either men or women (Table 2).

**Table 2:** General Health Among 1,880 Adults by Sex According to Tobacco Use in Sousse, Tunisia, 2009

Health status	Men			Women		
	Smoker, n (%)	Nonsmoker, n (%)	*P* Value	Smoker, n (%)	Nonsmoker, n (%)	*P* Value
Assesses health status as good	202 (74.5)	187 (66.8)	.04	485 (50.4)	18 (56.3)	.51
Often felt depressed during the previous month	158 (46.6)	111 (33.2)	<.001	23 (63.9)	480 (41.7)	.01
Often had little pleasure in doing things during the previous month	148 (43.7)	99 (29.6)	<.001	22 (59.5)	471 (40.8)	.02
Felt under a lot of pressure during the previous month	73 (21.5)	54 (16.2)	.07	13 (35.1)	285 (24.7)	.15

Among men, the proportion of alcohol consumption was significantly higher among smokers than among nonsmokers (25.3% vs 5.7%, *P *<.001) (Table 3). Concerning eating habits, male nonsmokers consumed more fruit and vegetables (45.6% vs 37.9%, *P* = .04) and avoided frequent consumption of high-fat, high-salt, and high-sugar foods (56.0% vs 42.9%, *P* = .001). Male smokers were more likely than nonsmokers to add salt to their food after it was cooked or when it was served (23.8% vs 15.6%, *P* = .007). Male smokers did less physical activity than did nonsmokers, but this difference was not significant. The proportion of obesity and abdominal obesity was significantly higher for male nonsmokers than for male smokers (Table 3). Differences between women smokers and nonsmokers with regard to these variables were not significant.

**Table 3:** Other Chronic Disease Risk Factors According to Tobacco Use Status Among 1,880 Adults in Sousse, Tunisia, 2009

Risk Factor	Men			Women		
	Smoker, n (%)	Nonsmoker, n (%)	*P* Value	Smoker, n (%)	Nonsmoker, n (%)	*P* Value
Consumes alcohol	86 (25.3)	19 (5.7)	<.001	NA	1 (0.1)	NA
Consumes 5 fruits and vegetables per day	129 (37.9)	152 (45.6)	.04	16 (43.2)	549 (47.8)	.58
Avoids eating high-fat, high-salt, or high-sugar foods	146 (42.9)	187 (56.0)	.001	21 (56.8)	621 (54.0)	.73
Adds salt to food after it has been cooked or when it is served	81 (23.8)	52 (15.6)	.007	11 (29.7)	254 (22.1)	.27
Does at least 150 min per week of moderate level of physical activity	145 (42.6)	154 (46.1)	.36	18 (48.6)	681 (59.1)	.20
Obese	49 (15.0)	70 (21.4)	.03	7 (19.4)	381 (33.7)	.07
Abdominal obesity	55 (16.6)	76 (23.2)	.03	23 (62.2)	706 (61.9)	.97
Hypertension	109 (33.5)	130 (40)	.08	10 (27.0)	344 (30.1)	.68

Abbreviations: NA, not applicable because no women smokers in the survey reported consuming alcohol.

## Discussion

The prevalence of tobacco use is high in Tunisia but few data are available on the association of this chronic disease risk factor with other lifestyle risks (3). The current project was conducted to investigate the association between smoking and other chronic disease risk factors among adult Tunisians to guide a community-based intervention to reduce tobacco use. A cross-sectional study was chosen that included evaluation of tobacco-use behaviors and knowledge.

Smoking prevalence found by our study was similar to that observed by previous Tunisian studies (19) (where more than half of men and 8% of women were current smokers) and akin to similar populations such as that of Morocco (20). In fact, the overall prevalence of current smoking in Morocco (20) was 18.0% (95% confidence interval [CI], 17.2–18.8): 31.5% (95% CI, 30.2–32.9) among men and 3.3% (95% CI, 2.8–3.8) among women in 2006. In Morocco, the prevalence of current smoking was inversely associated with level of education in men and increased with educational level in women. The risk of being a current smoker, compared with being a former or never smoker, was higher among lower educational groups (21).

A similar study of a Mediterranean population showed a direct association between smoking and serum triglycerides, intake of saturated fatty acids (by gram and percentage of total energy intake), and intake of dietary cholesterol. An inverse association was observed for smoking and intake of unsaturated fatty acids (percentage of energy intake), vitamin C, α-tocopherol, and β-carotene; leisure-time physical activity; and high-density lipoprotein cholesterol. These results suggest that smokers consume less fiber and several antioxidant components of the Mediterranean diet than do nonsmokers; smokers also show an adverse lipid profile (22). This finding underscores the importance of understanding differences in dietary patterns by smoking status for nutritionists and health educators involved in helping people make healthy dietary and lifestyle choices. These findings are consistent with those from high-income countries. For example, a study of noninstitutionalized adults aged 18 to 65 years (n = 1,543) who participated in the Food Habits of Canadians Survey (1997–1998) found that smokers consumed significantly fewer fruits and vegetables than did nonsmokers, leading to lower intakes of folate and vitamin C. The unhealthful diet of smokers places them at higher risk for chronic disease. Diet may act as a confounder in smoking–disease relationships (7).

Another study evaluated health-related quality of life and health risk behaviors among smokers (16). This study found that current smokers had significantly poorer health-related quality of life than did those who never smoked and were more likely to drink heavily, to binge drink, and to report depression and anxiety symptoms. Additionally, current smokers were significantly more likely than those who never smoked to be physically inactive, to report frequent sleep impairment, to report frequent pain, and to eat fewer than 5 servings of fruit and vegetables per day (16). In our study, we found that smokers more frequently than nonsmokers reported that they often feel depressed and have little pleasure in doing things. However, contrary to expectations, more smokers than nonsmokers reported that they have good health. 

This last finding suggests that smokers may be more likely than nonsmokers to underestimate their relative risk. A study showed that smokers believed their own risk of developing lung cancer is lower than the risk they estimated for smokers in general (23). Among current smokers, 44% considered that smoking can cause cancer only if daily smoking is higher than their own level of smoking, and an additional 20% considered that the cancer risk becomes high only if they smoke for more years than they have smoked already. Most smokers also agreed with other “risk denial” statements (eg, “smoking is not more dangerous than air pollution,” “some people smoke their whole life but never get sick”) (23).

More generally, there is an urgent need to better understand how smokers shape their risk denial because self-exempting beliefs may deter them from quitting their smoking habit (24). Furthermore, their perceived risk of lung cancer and of cancer in general barely increases with the number of cigarettes smoked per day, and their estimates of their overall risk of cancer are actually slightly lower than their estimates of their risk of lung cancer. Substantial proportions of smokers and former smokers agree with several myths; for example, more than half agree that exercise undoes most smoking effects (25). Given the accumulated evidence, the argument that people begin to smoke or continue to smoke with adequate knowledge of the potential risks appears indefensible (25). The need to improve smokers’ knowledge about their risk of developing chronic diseases is important. This need may be especially acute in many low- and middle-income countries.

Promotion of smoking cessation is an important goal to establish in Tunisia. Smoking cessation is a priority for preventing smoking-attributable disease and reducing its burden (26), including reducing risks of stroke, cardiovascular disease, and smoking-related cancers (27). Quitting smoking by the age of 30 could reduce the risk of dying from tobacco-related diseases by approximately 90% (28). Moreover, the clustering of multiple risk factors with tobacco use suggests that community-based interventions are needed to prevent tobacco use with the implementation of validated actions such as those described in the principles of World Health Organization Framework Convention on Tobacco Control.

To our knowledge, this study is the first to analyze the relationship between smoking and other lifestyle risk factors for chronic disease in Tunisia. Lifestyle habits such as tobacco use, physical inactivity, and unhealthy dietary practices were self-reported by the participants without objective evaluations of these variables. This lack of evaluation could have introduced a social desirability bias. To minimize this bias, standardized data collection procedures were followed. Reassurances were provided to put the participants at ease in the interview process, and they were assured that their data would remain confidential. Admittedly the percentage of female smokers in our study was small, but this is because the prevalence of smoking among women in Tunisia is low (29).

These results indicate that in addition to implementing smoking-cessation programs among patients who smoke, physicians and other health professionals should view smoking as a marker of an array of health-risk behaviors. In addition to prompting assessment of both mood and alcohol consumption, the results of this investigation suggest that our findings on physical activity, dietary habits, and mental health increase the importance of preventing tobacco use and may be important areas for assessment and potential targets for intervention among patients who smoke. Policy makers in Tunisia should implement a multisectoral program to prevent tobacco use and other chronic disease risk factors.
